# Contributing Factors to the Improvement of International Students' Health Literacy in China: A Self-Determination Theory Perspective

**DOI:** 10.3389/fpubh.2020.00390

**Published:** 2020-08-14

**Authors:** Fuli Zheng, Pingying Hu, Zhudan Lian, Yuan-Liang Wang, Siying Wu, Huangyuan Li

**Affiliations:** ^1^Department of Preventive Medicine, School of Public Health, Fujian Medical University, Fuzhou, China; ^2^School of Humanities, Fujian University of Technology, Fuzhou, China; ^3^Overseas Education College, Fujian Medical University, Fuzhou, China; ^4^Department of Epidemiology and Health Statistics, School of Public Health, Fujian Medical University, Fuzhou, China

**Keywords:** international students in China, health literacy, self-determination theory, need satisfaction, medical education

## Abstract

**Background:** It is generally accepted that learning engagement is predictive of better learning outcomes. Yet, there might be some underlying motives for students to engage in or disengage from learning.

**Aims:** Grounded in self-determination theory, this study aimed to examine whether satisfaction of international students' innate needs for autonomy, competence, and relatedness correlated positively with their engagement in learning and improvement of health literacy in China.

**Sample:** Forty-three international undergraduates from a medical university in China participated in the study.

**Methods:** Both qualitative and quantitative methods were used to deal with data collected from surveys on health literacy, perceived need satisfaction and the need satisfaction intervention, and from observation log recording dynamic changes in the students' performance while implementing a need-satisfying scheme in Hygiene education. In addition, final examination scores of with/without-intervention parts were compared to unveil the effect of the intervention.

**Results:** Perceived autonomy support motivated the participants to engage actively in learning; close relation to peers and teachers encouraged them to take on challenges; satisfying their need for competence enabled them to have better performance and academic achievements as well as an improvement on health literacy.

**Conclusions:** The present study suggested that fulfillment of the students' basic needs contributes to their engagement in learning and improvement of health literacy.

## Introduction

The goal of medical education is to cultivate creative medical talents with occupational competencies. Hygiene is an important module for all students majoring in Bachelor of Medicine and Bachelor of Surgery (MBBS) for international students in China. Since the teaching objectives and requirements of Hygiene are highly consistent with the basic knowledge and skills covered in health literacy, the “Scale of Health Literacy” introduced by the Chinese government is used as an index for evaluating students' learning outcomes of Hygiene. The concept of “health literacy” was first proposed in a report on health education and social policies (1). The World Health Organization defines health literacy as basic health information and services that individuals acquire, understand, and process, to make appropriate decisions in order to maintain and promote their own health (2). More and more international students are pursuing higher medical education in China, especially those from the “Belt & Road” countries. It is a great challenge for medical teachers to educate and train the international medical personnel from different countries. It seems that no pedagogy works well-enough with all the international students due to their diverse cultures and backgrounds. Popular teaching styles in China, such as lecture delivery, do not attract the international students. Some of them even skip classes and give up study. To achieve optimal learning outcomes, incentives and motivations are needed for students to learn actively and effectively.

It is generally accepted that learning engagement is predictive of better achievements. Yet there might be some underlying motives for students to engage in or disengage from learning. Pintrich (3) proposed that to further our understanding of students' motivation, we need to identify learners' needs that define what individuals want. In the self-determination theory [SDT; (4, 5)], innate needs for autonomy, competence, and relatedness are vividly termed as essential “psychological nutriments” for human beings to develop and function well (5). From the perspective of SDT mini-theory, also known as basic psychological needs theory [BPNT; (6)], learners could be motivated to regulate their learning behavior positively when their basic psychological needs are satisfied. That is, engagement in learning happens only when a student has autonomy over his own learning, feels competent in his learning, and experiences relatedness with his peers, teachers and important others. Medical students with autonomous motivation tend to: involve actively in learning, interact constantly with environment, integrate learning with practice, and invest in continuing professional development (7). Thus, the purpose of this study aims to examine whether satisfaction of learners' basic psychological needs in Hygiene education contributes to their learning outcomes, on which little research has been conducted.

## Methods

A need-satisfying intervention was implemented in this study, integrating task-based learning [TBL, (8, 9)], problem-based learning [PBL, (10)], and flipped classroom strategy (11) into Hygiene education for one semester to the meet diverse needs of the international students. Based on the integrated approach, the study investigated the contributing factors to the improvement of international students' health literacy in China.

### Hypotheses

Based on the SDT and the aim of the study, three hypotheses were formulated as follows:

H 1: The integrated approach could facilitate satisfaction of the students' basic needs for autonomy, competence, and relatedness.

H 2: Fulfillment of the students' basic psychological needs would motivate them to actively engage in learning Hygiene.

H 3: Learning engagement in Hygiene education would lead to good academic achievement and improved health literacy.

### Participants

The participants in this study involved two teachers and 43 international undergraduates from a medical university in China. Twenty-two of them were from “Belt & Road” countries, 21 from non- “Belt and Road” countries. All of them enrolled in 2015 and had studied in the university for two and a half years.

### Instruments

#### Scale of Health Literacy (CHLSM-E)

The evaluation scale on health literacy used in this study was the Chinese Health Literacy Scale for Medical Students in English, short for CHLSM-E (Supplementary 1). In order to collect valid data, the *Chinese Citizens' Health Literacy: Basic Knowledge and Skills (Trial)* was designed like a questionnaire to test the health literacy of Chinese college medical students by Wang (12). In this study, it was translated into English, piloted, and modified for better understanding. The generated CHLSM-E was first tested on 2014 MBBS students for the elimination of potential ambiguity. The validity of CHLSM-E was then confirmed by the expert assessment method and Kaiser-Meyer-Olkin test (0.865). The reliability was assessed by Cronbach's test (α = 0.772). The subjects were required to choose right answers to the 68 questions, the total score of the scale is 90.

#### Need Satisfaction Scale

The formally validated instrument “the Basic Psychological Need Satisfaction and Frustration Scale” (13) was used to measure the subjects' perceived satisfaction of the three psychological needs. The student participants were required to respond to a 5-point Likert scale ranging from 1 (not at all true) to 5 (very true) to indicate the degree to which the statement was true for them.

#### Post-intervention Questionnaire

After implementation of the need-satisfying intervention, a post-intervention questionnaire was distributed to gather feedback upon the module. The questionnaire includes 13 questions, containing active engagement, study interests, attitude to group study and comparison to traditional lecture strategies.

#### Interviews

To better understand the respondents' perceived need satisfaction and its relationship with the integrated approach used in the intervention, semi-structured interviews were conducted. The interview covered eight questions, including their perceived satisfaction of three basic needs, integrated learning model, teacher support, teamwork, flipped classroom, multi-dimensional assessment of learning outcomes. Fifteen randomly chosen student participants were interviewed. Views with similar meanings from 10 or more students were considered as common and typical views and listed in **Table 2**.

#### Observation Journals

Observation journals were kept by the 2 teachers while implementing the need-satisfying scheme in Hygiene education. Data collected from the observation journals recorded the students' learning performance, providing evidences for assessment of the students' learning outcome.

### Intervention

The need-satisfying intervention was carried out with an integrated approach, involving 5 phases. Firstly, the class with 43 international students were divided into several groups with mixed nationalities. Each group chose a Hygiene content-based topic with key questions designed by the teachers. Secondly, group members must prepare a topic-based “lesson” for their classmates. They had the freedom to use materials provided by the teachers or resources they preferred. Students studied together in groups and divided the task into pieces, taken into account each individual's strengths, interests, and personality. Thirdly, the group members gave the “lesson,” clarified confusion, and responded to challenging questions from peers. All kinds of presentation formats were accepted. This was followed by the multidimensional assessment of their performance and suggestion for improvement from teachers, peers and the group members. Lastly, the teacher concluded by going through key points of the lesson. The intervention was performed for environmental health, which covers approximately half of the semester. The rest of the module (i.e., occupational health) was taught by traditional lectures.

### Data Collection

**Figure 1:**
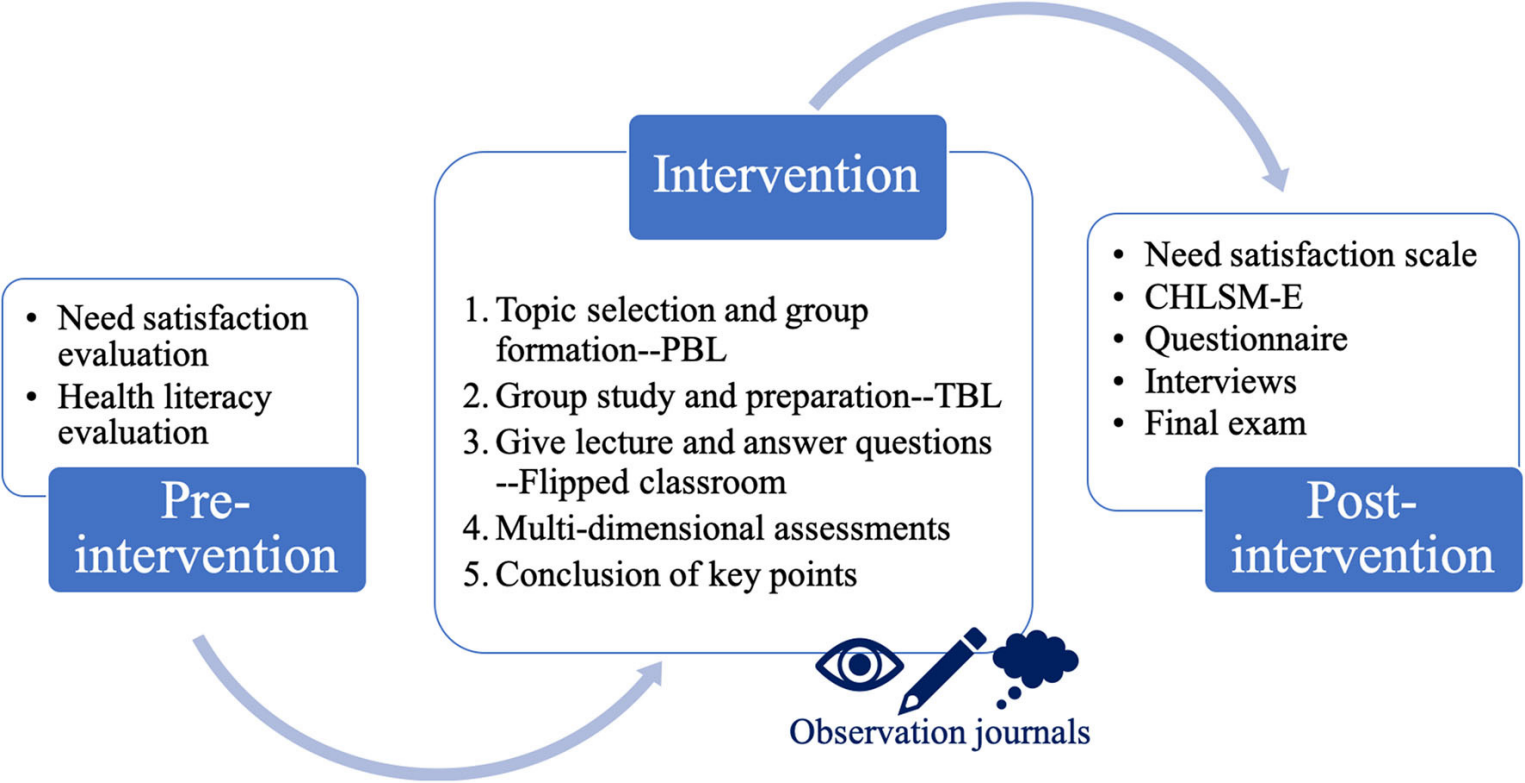
Study design flowchart. Before intervention, students' need satisfaction and health literacy were evaluated by the Basic Psychological Need Satisfaction and Frustration Scale and the Chinese Health Literacy Scale for Medical Students in English (CHLSM-E), respectively. During 5-phase-intervention, integrated approach was used to fulfill students' need satisfaction and promote active engagement in learning. During the intervention, observation journals were kept by teachers for documentation, assessment and reflection. After the intervention, not only need satisfaction and health literacy were evaluated for comparison, but also did we collected data from interviews, questionnaire and final exam. The outcome of this study was solidified by quantitative and qualitative analysis.

The researchers conducted surveys regarding health literacy and need satisfaction and collected data in regular classes. Perceived change in satisfaction of psychological needs and levels of health literacy of the participants were measured via questionnaires before and after the intervention, so that growth and improvement could be calculated. Learning outcome was also confirmed by comparing final exam scores with/without intervention. Moreover, qualitative data were collected from interviews and observation journals to evaluate the integrated approach used in the need-satisfying intervention. The study design flow chart is shown in Figure 1.

### Data Analyses

Data collected from pre- and post-questionnaires were processed and analyzed with SPSS 22.0. A two-way ANOVA followed by Sidak's multiple comparisons test was performed to identify the improvement of the students' health literacy. Data gathered from interviews and observation journals were coded by summarizing some typical, common and convincing comments. The quantitative statistics and the qualitative data supported each other to verify the research hypotheses.

## Results

This study yielded sufficient data that provided valuable insights into the perception and practice of teaching international medical students in China.

### Results of the Surveys

Two sets of data were collected from the surveys to measure changes in the students' perceived need satisfaction and level of health literacy before and after the intervention.

#### Growth of Perceived Need Satisfaction and Promotion of Active Engagement

Results of pre- and post-questionnaires about need satisfaction using the need satisfaction scale were listed and compared in Table 1.

**Table 1:** Perceived need satisfaction.

**Subscales**	**Pre-questionnaire**			**Post-questionnaire**		
	**α**	**Mean**	**SD**	**α**	**Mean**	**SD**
Need for autonomy	0.773	3.03	0.531	0.797	4.58	0.341
Need for competence	0.851	3.34	0.542	0.863	4.36	0.416
Need for relatedness	0.832	3.19	0.625	0.825	4.42	0.485

Table 1 shows the growth of the respondents' perceived need satisfaction, which proved that the integrated learning approach played a positive role in meeting the international students' psychological needs for autonomy, competence, and relatedness.

According to the feedback from post-intervention questionnaire, 65 and 22.5% of students declared that the intervention helped provoke learning interests effectively and very effectively, respectively. Besides, 87.5% students believed that the intervention effectively helped cultivating autonomous learning skills. Collaborating to the questionnaire result, students creatively integrated various formats in demonstrating key topics such as interviews, self-directed videos, DIY purification instruments, and role-play for goiter diagnosis. Consistently, 55% of students scored their peers' presentation as quite good, and 37.5% scored wonderful/ excellent. The satisfactory feedback suggested that not only the ones presenting, but also their peers, the ones participating, were fond of the learning process. Follow-up interviews were conducted to collect data concerning basic need satisfaction after the application of the integrated approach in the intervention. Common and typical views about basic psychological need satisfaction were summarized and compared in Table 2.

**Table 2:** Corroborating information gathered from interviews.

**Having autonomy**	**Feeling competent**	**Feeling relatedness**
1. We are allowed to choose tasks and learning styles we are interested in and skilled at. 2. Autonomy support motives us to engage actively in self-directed and collaborative learning and preparation for flipped classroom. 3. Each group are offered a chance to do a presentation for classmates on the chosen topic in flipped classroom. We are driven to take on the challenge.	1. Teacher and peer supports make us feel competent to challenge problems and work out solutions. 2. We have prepared well and confident to present what we have learned about the chosen topic to classmates. 3. We are able to tackle problems raised by peers and teachers.	1. Positive comments and valuable feedback from teachers and peers encourage us to move on. 2. Teamwork makes arduous tasks manageable and enjoyable. 3. Care and concern for each other inspires our enthusiasm for learning activities.

These results suggested that autonomy motivated the students to engage actively in learning tasks. Students stated that collaborative learning and support from teachers made arduous tasks manageable and enjoyable. They felt competent to challenge problems and to work out solutions. They appreciated positive comments and valuable feedback from teachers and peers, which inspired their enthusiasm for flipped classroom activities. These data were in line with the results from the questionnaire. Both quantitative and qualitative data supported each other to prove the first two hypotheses that the integrated learning approach facilitated satisfaction of the student participants' basic needs for autonomy, competence, and relatedness, which motivated them to engage in learning actively.

#### Improvement of Health Literacy

Results of pre-test and post-test of the international medical students' health literacy were listed and compared below (see Table 3).

**Table 3:** Results of health literacy.

**Subscales**	**Pre-test (*****N*** **=** **43)**		**Post-test (*****N*** **=** **43)**		***t***	***P***
	**Mean**	**SD**	**Mean**	**SD**		
Healthy lifestyle	34.70	8.531	43.99	7.314	6.280	<0.0001
Basic knowledge	24.24	6.542	29.08	4.921	3.272	0.0047
Basic skills	9.55	7.625	13.86	5.845	2.913	0.0152
Total score	68.49	7.366	86.93	6.048	12.46	<0.0001

Comparing the mean values of pre-test and post-test, we can see that the international students made great progress after the need-satisfying intervention in Hygiene education for one semester. Fittingly, the ANOVA analysis of pre-test and post-test also confirmed that their progress in health literacy significantly improved in three subscales of health literacy and in the total score (*P* < 0.05). Furthermore, we analyzed students' final exam scores in detail by comparing the groups with- or without-intervention. After converting the portion scores into percentages, the group with-intervention scored 80.47 ± 7.20, while the group without-intervention scored 76.33 ± 13.41 (*P* < 0.05). The results supported the third hypothesis that engagement in learning in Hygiene education would lead to good academic achievement and improved health literacy.

### Supporting Evidences From Observation Journals

An observation journal contains five components: date, topic and content, positive effect, problems or weaknesses, and suggestion for improvement. The observation logs showed that the students became more and more active and enthusiastic as the intervention proceeded in presenting their learning outcomes, interacting with peer presenters, questioning, providing encouraging feedback and assessment for classmates. These supporting evidences corroborate the second hypothesis that fulfillment of the students' basic psychological needs would motivate them to engage in active learning, which contributed to good performances. Some typical comments are quoted as follows.

Sep. 25, 2018

*It seems that the students are more proactive in learning what they are interested in. Obviously, the students bring their potential into full play to learn when they are allowed to make their own decision and choose what they like*.

Oct.16, 2018

*I enjoy observing students' well-prepared and creatively-presented group work. The innovative elements and their special way of presenting often arouse laughter, and attract peers' attention. It seems that teamwork and supports from teachers make them feel competent to challenge problems and work out solutions*.

Dec. 20, 2018

The peer presentations give me big surprises from time to time.

*They perform very well so that their classmates and teachers are fascinated and interact enthusiastically*.

## Discussion

### Mechanism of Improving Health Literacy

**Figure 2:**
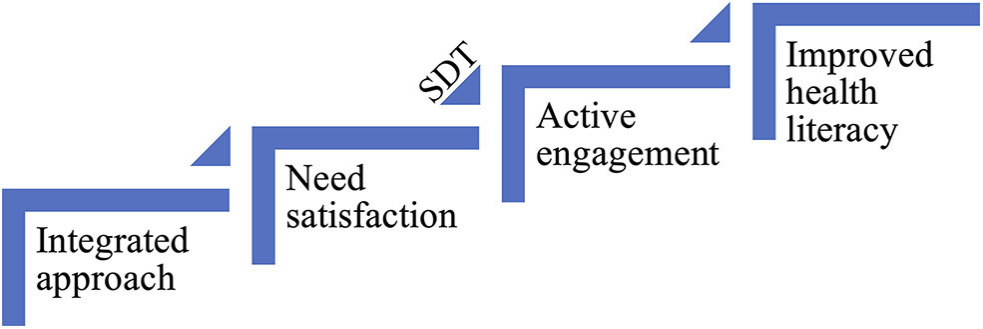
The mechanism of improving health literacy. Pedagogical strategies such as task-based learning, problem-based learning and flipped classroom were integrated in Hygiene education to meet the students' basic psychological needs for autonomy, competence and relatedness. According to self-determination theory (SDT), once their basic needs were satisfied, they actively engaged in learning. As a result, the students' health literacy was improved.

In this study, quantitative and qualitative data supported each other to illustrate the dynamic mechanism for improving health literacy of international medical students in China. Based on learner-centered principles, an integrated learning and teaching approach was designed and used in Hygiene education. The integrated approach helped meet the students' basic psychological needs for autonomy, competence, and relatedness. Satisfaction of the innate needs motivated the students to engage in learning actively, which contributed to satisfying learning outcomes and improved health literacy (Figure 2).

### Contributing Factors to Learning Engagement

In the study, a need-satisfying intervention was implemented in Hygiene education. In the intervention, the students were offered freedoms to choose team members (of mixed nationalities), goal-oriented tasks, learning resources, learning style and method, and so on. The students' perceptions of autonomy from their instructors led to an increase in self-regulation, perceived confidence in the subjects, and a decrease in anxiety regarding course grade (14). In addition, since each group was supposed to do a presentation on their chosen topic together, the students revealed that teamwork and support from the teachers make arduous tasks manageable and enjoyable. They felt competent to challenge problems and to work out solutions, which also proved that interactive and collaborative learning could effectively stimulate learning motivation (15). Lastly, positive feedback on their work from teachers and peers encouraged connection and inspired their enthusiasm for learning. Green-Demers and Pelletier (16) found that when peers and teachers fostered relatedness by providing affiliation and interpersonal support, this way, students were more committed to academic endeavors, which in turn enhanced their overall well-being. Conversely, Legault et al. (17) demonstrated that a lack of interpersonal support was significantly associated with motivational issues such as having difficulty in internalizing the importance of academic activities and having trouble in developing and sustaining motivation at school.

### Approach Leading to the Main Findings

Results of health literacy tests validated the effectiveness of the integrated approach used in the intervention. Task-based learning (TBL) facilitated the students to achieve learning outcome by completing learning tasks fused with targeted Hygiene knowledge and skills (8, 9). Each task consisted of group learning and presentation, focusing on the three key questions and problems provided by teachers. The key questions and problems of each task were designed so as to meet the learning objectives of each topic. These questions and problems motivated and directed the students to search for information and find solutions. The problem-based learning (PBL) approach contributed to the acquisition of knowledge and skills in the process of solving problems (18). Moreover, the flipped classroom model (11) drove the presenting students to acquire relevant knowledge before class via self-directed learning (SDL). Students also collaborated in the classroom by presenting a “lesson” and participated in discussion. In other words, this goal-oriented integrated approach involved in TBL, PBL, SDL, collaborative learning, flipped classroom, and practice according to contents and learners' preferences. The application of the integrated approach met the diverse needs of the students and motivated them to get involved in learning, thereby producing satisfying results.

### Application of CHLSM-E

There were a variety of evaluation tools for measuring health literacy, such as the widely used TOFHLA (Test of Functional Health Literacy in Adults) (19) and REALM (Rapid Estimate of Adult Literacy in Medicine) (20). In addition, some tools are focused on specific context such as cancer (21), blood pressure (22), dental health (23), diabetes (24), mental health (25), and so on, but none of the existing scales were available and suitable for international college medical students studying in China. Therefore, we formulated our own scale of health literacy to fit our need, which suited the conditions unique to China and the international backgrounds of the students. This practice was similar to that of some previous researchers who modified and translated the available scales into their native languages, such as into Arabic (26), Spanish (27), Japanese (28), and French (29) to suit their needs. The CHLSM-E provides an alternative tool for international researchers to evaluate health literacy level of young adults, especially college medical students. More importantly, for the first time, we translated the Chinese literacy scale into English. This adds an important component to the field of health literacy. It would be interesting to compare the similarities and differences of the definitions and contents among eastern and western perspectives.

### Future Directions

Based on an integrated approach to studying the simultaneous relationship between basic innate needs and social-cognitive influences, this study was structured to demonstrate the relationship between basic psychological needs and learning outcome. However, there are still some limitations that require further improvements. For future research, more students could be involved in the study. Moreover, the students should be randomly divided into two groups for comparative study, with one group employing the integrated approach and the other using the traditional teaching method. Furthermore, the online interactive platform should be set up for discussion and forums so that students could benefit from interactive learning.

In conclusion, the CHLSM-E can be used to evaluate learning outcomes of the Hygiene class for international medical students in China since the teaching objectives of Hygiene are in line with the evaluation index of college medical students' health literacy. The integrated learning and teaching approach not only met the internationals students' diverse learning needs in medical education in China but also fulfilled their basic psychological needs for autonomy, competence, and relatedness. The approach was efficient in improving their learning outcome. Therefore, teachers should never limit students' imagination, creativity and competencies but encourage them to tackle problems and find solutions. Moreover, teacher support and teamwork make tasks manageable and enjoyable. This study provides a paradigmatic intervention for medical education of international students in China. It also provokes pedagogical reform and innovation in other courses.

## Data Availability Statement

The raw data supporting the conclusions of this article will be made available by the authors, without undue reservation.

## Ethics Statement

The studies involving human participants were reviewed and approved by the ethics committee of Fujian Medical University. The patients/participants provided their written informed consent to participate in this study.

## Author Contributions

FZ designed and conducted the intervention, analyzed the data, and drafted the manuscript. PH helped with the generation of hypothesis, designed the intervention, and revised the manuscript. Y-LW helped with the revision of the manuscript. ZL, SW, and HL helped with the design of the study and the interpretation of data. All authors contributed to the article and approved the submitted version.

## Conflict of Interest

The authors declare that the research was conducted in the absence of any commercial or financial relationships that could be construed as a potential conflict of interest.
