# Supplementary material for: Contributing Factors to the Improvement of International Students' Health Literacy in China: A Self-Determination Theory Perspective
Source: Front Public Health. 2020 Aug 14;8:390. doi: 10.3389/fpubh.2020.00390 (PMC7456880; doi:10.3389/fpubh.2020.00390)
Supplement: Supplementary file 1 [file Data_Sheet_1.DOCX]

Supplementary Material

Supplementary 1. Chinese Health Literacy Scale for Medical Students in English (CHLSM-E)

Evaluation Scale on Health Literacy

Dear students,

This questionnaire concerns about the health literacy of international students in China, which includes your capabilities to acquire health knowledge, information and service, as well as your understandings of healthy lifestyle and health skills. Your participation is very important to our research.

The responses and private information will be handled **strictly confidential** and **only** used for our research. The outcome of this study will provide important evidence for the evaluation of health literacy levels of international students in China and for the modifications of teaching contents in Module Hygiene. Thank you in advance for your support and cooperation.

**Section 1. Personal information (Please choose the one that suits you the most).**

1. Your age range:

①21 or below

②22-24

③25-27

④28-30

⑤31 or above

2. Your gender:

①Male

②Female

③Non-binary

3. Your grade:

①Freshman (first year)

②Sophomore(second year)

③Junior (third year)

④Senior(fourth grade)

⑤Fifth grade

⑥Sixth grade

⑦Seventh grade

4. Your Nationality:

①China

②India

③Thailand

④The United Republic of Tanzania

⑤Nigeria

⑥Zambia

⑦Zimbabwe

⑧Bangladesh

⑨Ghana

⑩Others

5. Where are you from?

①City

②Urban area or rural area

6. Are you a single child in your family?

①Yes

② No

7. What is your father’s education level?

①Elementary school and below

②High school

③College and University

④Postgraduate

8. What is your mother’s education level?

①Elementary school and below

②High school

③College and University

④Postgraduate

9. What’s the monthly income of your family?

①Less than 1000 Yuan

②1000～5999 Yuan

③6000～9999 Yuan

⑤10000～14999 Yuan

⑥More than 15000 Yuan

10. How much is your monthly living expenses?

①Less than 800 Yuan

②800～2000 Yuan

③2001～4000 Yuan

④4001～6000 Yuan

⑥>6001Yuan

11. How will you evaluate your health condition?

①Very good

②Good

③Not bad

④Bad

⑤Very bad

**Section 2. Questions about health**

What kind of disease can flies, mice, mosquitoes and cockroach (black beetles) spread, respectively?

|  | Pestilence | Malaria | Dysentery | Not sure |
| --- | --- | --- | --- | --- |
| 12. Flies | ① | ② | ③ | ④ |
| 13. Mice | ① | ② | ③ | ④ |
| 14. Mosquitoes | ① | ② | ③ | ④ |
| 15. Cockroach | ① | ② | ③ | ④ |

People have their own opinions on ‘health’. What’s your opinion about the following statements? Please choose the one that suits you the most.

|  | Agree | Neither agree nor disagree | Disagree | Not sure |
| --- | --- | --- | --- | --- |
| 16. Health means being in good physical condition with no disease. | ① | ② | ③ | ④ |
| 17.One in good health physically does not need to pay attention to health problems. | ① | ② | ③ | ④ |
| 18.I will pay attention to my health when I have money. | ① | ② | ③ | ④ |
| 19.Prevention goes first: Choosing to live a healthy life as soon as possible is a good investment. | ① | ② | ③ | ④ |

20. If being scratched or bitten by cats and dogs, what would you do?

① Vaccinate against rabies immediately

②Wash the wound with soap and water, then vaccinate against rabies as soon as possible

③Wash the wound with water is enough

④Treat the wound in traditional ways

⑤Not sure

21. An adult’s normal pulses should be?

①30～50 beats/min

②60～100 beats/min

③100～120 beats/min

④Not sure

22. How many milliliters (ml) of blood are appropriate for blood donation of an adult?

①Less than 200 ml

②200～400 ml

③400~600 ml

④Not sure

23. Which option is the normal axillary temperature of an adult?

①35～36℃

②36～37℃

③37～38℃

④Not sure

24. Which option is in the range of normal blood pressure (systolic pressure/ diastolic pressure)?

①140/95mmHg

②105/75mmHg

③150/100mmHg

④Not sure

25. When symptoms such as coughing and expectoration (spitting out) developed for more than two weeks or symptoms such as hemoptysis (spitting blood), low fever, weakness, night sweat appeared, which of the following is the best thing to do?

①Have some rest and improve nutrition

②Get some cold medicine

③Have symptomatic treatments

④See a doctor in TB control agency

⑤Not sure

26. In your opinion, how many hours of sleep should an adult have at least per day?

①5～6 hours

②7～8 hours

③9～10 hours

④Not sure

27. Do you agree with the following statement: Diseases are mainly caused by ourselves and have nothing to do with environmental pollution?

①Agree

②Neither agree nor disagree

③Disagree

④Not sure

28. Do you agree with the following statement: Since having injections works better than taking medicine, one should get injections whenever he/she is ill.

①Agree

②Neither agree nor disagree

③Disagree

④Not sure

29. Are you willing to work or study with the disabled and the recovered mental patients?

①Very willing to

②If needed, I won’t refuse

③Not willing to, and I’ll ask for switch of work

④No, never will I work with those people

⑤Not sure

30. Please choose the ones that can cause Schistosomiasis (blood flukes disease)?

①Eat unclean food

②Drink unboiled water

③Touch water or swim in the water contaminated with Oncomelania

④Not sure

31. Do you agree with the following statement: Dietary supplements cannot be used as medicine to treat an illness.

①Agree

②Neither agree nor disagree

③Disagree

④Not sure

32. Do you agree with the following statement: Employers must provide employees with protective equipment.

①Agree

②Disagree

③Not sure

33. In your opinion, which of the following is the most effective and economical way to prevent infectious diseases such as measles hives and hepatitis B?

①Enhance nutrition

②Do physical Exercises

③Breast-feed

④Receive vaccination

⑤Pay attention to personal hygiene

⑥Not sure

34. In your opinion, how can pulmonary tuberculosis be passed around?

① Through eating raw, cold or unclean food

② When patients with pulmonary tuberculosis cough, sneeze and talk loudly, droplets coming out can transmit the disease

③ Through blood transfusion

④ Though hand shaking or clothes touching

⑤ Not sure

35. Which of the following is the law specialized in protecting the rights of laborers who work with poisonous and harmful materials in China?

①Labor law

②Law of environmental protection

③Law of Occupational Disease Prevention and Treatment

④Not sure

**For the following questions (36-42), you can choose multiple answers.**

36. Opinions on ‘mental health’ vary from person to person. Please choose the opinions that you agree with.

①Everyone can come across mental health problems in various stages of one’s whole life.

②Mental health has much to do with interpersonal relationships.

③Mental problems are not diseases, thus there’s no need to see a psycho doctor.

④I have a strong mind, so I am in good mental health.

37. Do you think HIV can be spread in the following ways?

①Have meals with people living with HIV
②Have sex with people living with HIV
③Swim with people living with HIV
④Share shaving razor with people living with HIV

⑤Mosquito bites
⑥Transfuse blood and blood products infected by HIV

⑦Shake hands with people living with HIV
⑧Mother who infected with HIV passes it to the child by giving birth or breastfeeding

38. In your opinion, which of the following might be warning signals of cancer?

①Abnormal lumps

②Abnormal bleeding

③Unknown weight loss

④Generalized fatigue and weakness

⑤Not sure

39. If you found poultry or birds died of diseases, what would you do?

①Report to relevant authorities

②Bring home and fully cooked before eating

③Deeply buried on the spot

④Ignore

⑤Feed other animals with the dead bodies

⑥Throw them into a river

⑦Burn up the dead bodies

⑧Sell them

⑨Not sure

40. In your opinion, what does a healthy lifestyle include?

①Quit smoking and limit alcohol

②Have a balanced diet

③ Be Open minded

④Take more diet supplements and health products

⑤Control body weight

⑥Eat more and sleep more

⑦Exercise regularly

⑧Not sure

41. In your opinion, which of the following statements about giving up smoking are correct?

①The sooner, the better.

②As long as smoking is quit, (no matter when it is quit), it is good for health.

③One can never quit tobacco.

④There’s no need for the elderly to quit smoking. ⑤Not sure

42. In your opinion, in order to cure pulmonary tuberculosis, what should we do?

①Make early detection and early treatment

②Take medicine regularly during course of treatment

③Enhance nutrition

④Rest more

⑤Not sure

**Section 3. Daily behavioral habits**

43. Sharing towels and toothbrushes with families but not others.

①Totally agree

②Somewhat agree

③Disagree

④Not sure

44. The following statements are about sleeping pills and painkillers. Please choose the one that suits you the best.

①People can take sleeping pills according to their physical condition.

②Sleeping pills are not addictive.
③Painkillers are addictive and should not be taken casually.
④Not sure

45. There are some statements about antibiotics. Please choose the ones you think are/is right.

①Antibiotics should be taken as soon as we caught a cold.

②People can get antibiotics by themselves according to illness conditions.

③Antibiotics should be taken under a doctor’s guidance.

④Antibiotics can kill both bacteria and viruses.

⑤Not sure

46. In your opinion, what is the best way to eliminate indoor air pollution?

①Open windows frequently

②Spray disinfectants

③Spray air freshener

④Not sure

47. How many grams of milk or dairy products are appropriate for an adult per day?

①200 g

②300 g

③400 g

④Not sure

48. Which of the following order is the most reasonable daily intake, from the highest to the lowest?

①Grains> vegetables, fruits>meat, eggs and dairy products> fats

②Vegetables, fruits > grains > meat, eggs and dairy products> fats

③Meat, eggs and dairy products > vegetables, fruits > grains > fats

④Not sure

49. Which of the statements below about driving do you agree with?

①Seatbelt doesn’t need to be fastened when driving.

②Motorcyclists have to wear helmets.

③It is okay to drive when one only drinks a little alcohol.

④It is okay to drive overspeed if there’s less traffic.

⑤Not sure

50. Which of the following statement about the best-before date (expiration date) is correct?

①As long as the food still looks alright, it can still be consumed even it has passed the best before date.

②Never eat anything that has passed the best-before date.

③Food that has passed the best-before date can be still consumed after cooked or boiled.

④Not sure

51. In order to prevent high blood pressure, what is the maximal amount of salt an adult should intake per day?

①2 g

②6 g

③8g

④12 g

⑤Not sure

52. In your opinion, how many percentages exceed standard weight would be considered as overweight and obesity, respectively?

①5%, 10%

②5%, 15%

③10%, 15%

④10%, 20%

⑤15%, 25%

⑥Not sure

53. Which of the following statement is correct?

①The cutting board used for vegetables and raw meat should not be used for cooked meat and cold dishes.

②After rinsing with water, the kitchen knife used for vegetables can be used to chop cooked meat.

③The cutting board for raw meat can be used for cooked meat once it has been rinsed with water.

④Not sure

54. How many physical examinations should a pregnant woman get during her pregnancy?

①3 times②5 times③7 times④Not sure

**For the following questions, you can choose multiple answers.**

55. What diseases can be caused by passive smoking?

①Lung cancer

②Coronary heart disease

③Chronic Obstructive Pulmonary Disease

④Dentalcaries

⑤Gastroenteritis

⑥Cataract

⑦Erectiledysfunction

⑧Osteoporosis

⑨Not sure

56. If you are ill, what will you usually do?

①See a doctor in time

②Get some medicine from a pharmacy

③Take medicine according to the doctor’s advice

④Stop taking medicine once the symptoms disappear

⑤Visit the doctor subsequently according to the doctor’s advice

⑥Not sure

57. When purchasing and using pesticides, which of the following statement do you think is correct?

①Pesticides can be put in the same bag (or basket) together with daily necessities.

②Pesticides should be stored in a place far from children.

③Pesticides can be used empirically.

④When pesticides poisoning occurs, the first thing to do is to stimulate vomiting.

⑤Not sure

58. Which of the following diseases could be caused by water contamination?

①Hepatitis A

②Hepatitis B

③Poisoning

④Cancer

⑤Diarrhea

⑥Measles

⑦Not sure

59. If there’s sputum in the throat, what would you do?

①Swallow it

②Split on to the floor

③Split into a piece of handkerchief or tissue.

④Split into the toilet.

⑤Not sure

60. Which of the following statements about infants feeding are correct?

①Formula milk powder is more nutrient than breast milk

②Start breastfeeding the sooner the better

③Introduce solid foods when the child is six-month-old.

④Cereals/grains should be added first as solid food for children.

⑤Not sure

**Section 4. Questions about health skills**

61. Where should fingers place when measuring pulses?

①Measure at A ②Measure at B ③Measure at C ④Not sure


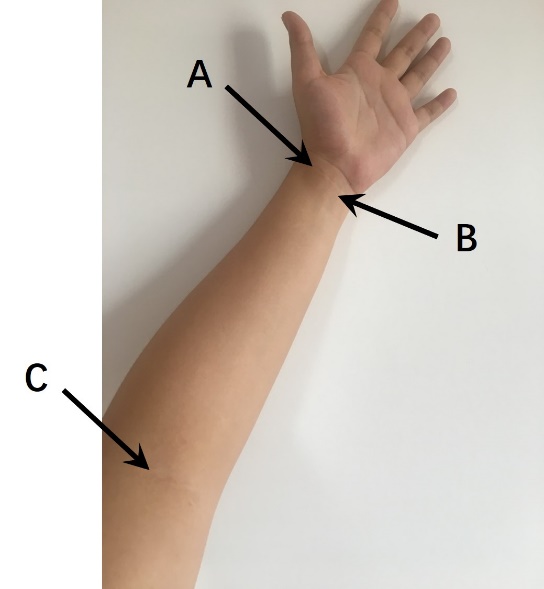


62. Please match the signs with their corresponding meanings one by one.

①Explosive, Toxic, Biohazard, Radioactivity.
②Explosive, Toxic, Radioactivity, Biohazard.
③Inflammable, Dangerous, Radioactivity, Biohazard.
④Inflammable, Toxic, Biohazard, Radioactivity.
⑤Explosive, Dangerous, Radioactivity, Biohazard.
⑥Not sure.


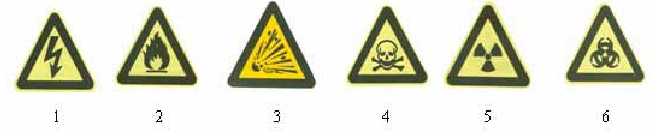


63. What is the meaning of OTC on the box of medicine?

①The medicine needs to be prescribed by a doctor.

②The medicine doesn’t need a prescription

③Not sure

64. Do you agree with the following statement: For first aid treatment on fracture, fracture reduction has to be processed first.

①Agree

②Disagree

③Not sure

**For the following questions, you can choose multiple answers.**

65. If you find someone is suffering carbon monoxide poisoning, what would you do?

①Open the window

②Move him/her to a well-ventilated place with fresh air

③Make emergency calls

④Move him/her to a cooler place

⑤Not sure

66. To rescue a victim from electric shock, we should:

①Pull and drag the victim directly

②Switch off the power immediately

③Separate the electric shocker and power by a dry crabstick

④Separate the electric shocker and power by a metal stick

⑤Not sure

67. When a fire breaks out, one should:

①Cover nose and mouth with wet towel

②Lie prostrate and evacuate from the fire ground

③Call 119 immediately

④Evacuate from the fire ground via elevator

⑤Put on clothes and bring valuables, then evacutate from the fire ground

⑥Not sure

68. What would you do if encounter someone with respiratory and cardiac arrest?

①Artificial respiration

②External chest compression

③Call 120 for an ambulance

④Call the police

⑤Not sure

** The correct answers are labelled in red.
